# Multiple subcutaneous nodules as the primary presentation of angioimmunoblastic T-cell lymphoma: a case report and literature review

**DOI:** 10.3389/pore.2026.1612446

**Published:** 2026-06-11

**Authors:** Ying Li, Yanxia Cai, Xiaoyan Xu

**Affiliations:** 1 Department of Pathology, Sanya Central Hospital (The Third People’s Hospital of Hainan Province), Sanya, China; 2 Department of Dermatology, Sanya Central Hospital (The Third People’s Hospital of Hainan Province), Sanya, China; 3 Department of Pathology, College of Basic Medicine of Inner Mongolia Medical University, Hohhot, Inner Mongolia, China

**Keywords:** angioimmunoblastic T-cell lymphoma, case report, cutaneous manifestation, lymphadenopathy, subcutaneous nodules

## Abstract

Angioimmunoblastic T-cell lymphoma (AITL) frequently involves the skin, typically presenting as rash or pruritus. However, AITL presenting primarily with multiple subcutaneous nodules is exceedingly rare and poses significant diagnostic challenges, often leading to misdiagnosis. We reported a 53-year-old man who presented with widespread subcutaneous nodules. He had a 1-month history of multiple painless subcutaneous nodules on the trunk and upper limbs. Physical examination revealed multiple firm nodules measuring 0.5–2 cm, accompanied by dark red plaques. Laboratory investigations showed mild anemia, elevated inflammatory markers, decreased IgA levels, and increased IgE levels. Imaging findings demonstrated generalized lymphadenopathy. Histopathological examination revealed effacement of the lymph node architecture with atypical lymphoid cells; immunohistochemistry was positive for CD3, CD4, PD-1, and EBER. A diagnosis of AITL of stage III was established. The patient achieved complete remission (CR) after 6 cycles of chidamide plus CHOP chemotherapy, with no evidence of recurrence at 6 months. This case broadens the recognized cutaneous spectrum of AITL and underscores the importance of considering lymphoma in the differential diagnosis of unexplained subcutaneous nodules.

## Introduction

Angioimmunoblastic T-cell lymphoma (AITL), also known as nodal T-follicular helper cell lymphoma (angioimmuno-blastic-type), is an aggressive subtype of peripheral T-cell lymphoma (PTCL) derived from T-follicular helper (TFH) cells. It accounts for approximately 2% of all non-Hodgkin lymphomas and 15%–20% of PTCLs and is characterized by prominent inflammatory and immune dysregulation [1]. Its main clinical manifestations include lymphadenopathy (LAP), B symptoms (e.g., fever, night sweats, weight loss) and hypergammaglobulinemia [2]. Additional features may include anemia, hemolysis and hepatosplenomegaly.

Approximately 50% of AITL patients also have cutaneous involvements, typically manifested as rash and/or pruritus [3], and are often misdiagnosed as drug eruption or viral exanthem. However, AITL presenting with multiple subcutaneous nodules as the primary manifestation is rare [4] and may be misdiagnosed as infectious skin diseases, connective tissue disorders, or metastatic carcinoma [5, 6]. Here, we report a case of AITL presenting primarily with multiple subcutaneous nodules in the absence of other typical cutaneous manifestations. This case highlights the importance of considering AITL in patients presenting with unexplained cutaneous nodules as primary symptoms.

## Case presentation

A 53-year-old man presented to our department in September 2025 with a 1-month history of multiple subcutaneous nodules, initially involving the back and subsequently spreading to the trunk and upper limbs. He denied pruritus or pain, as well as any history of tuberculosis, hepatitis, leprosy, or other infectious diseases, and reported no fever, night sweat or weight loss.

**FIGURE 1:**
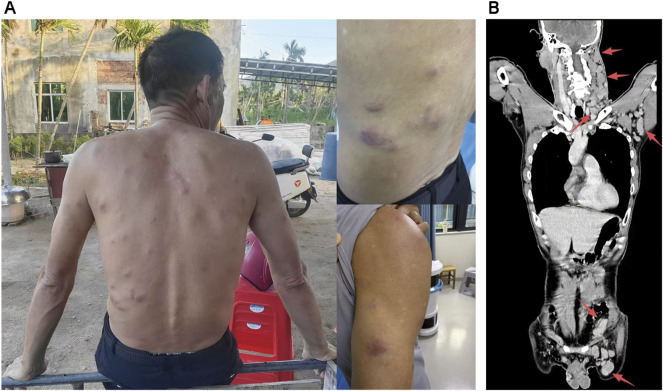
Clinical appearance and imaging findings before treatment. **(A)** Multiple subcutaneous nodules on the patient’s back, trunk and upper limbs. **(B)** CT imaging revealing diffuse lymphadenopathy involving cervical, supraclavicular, axillary, retroperitoneal, pelvic, and inguinal regions.

**FIGURE 2:**
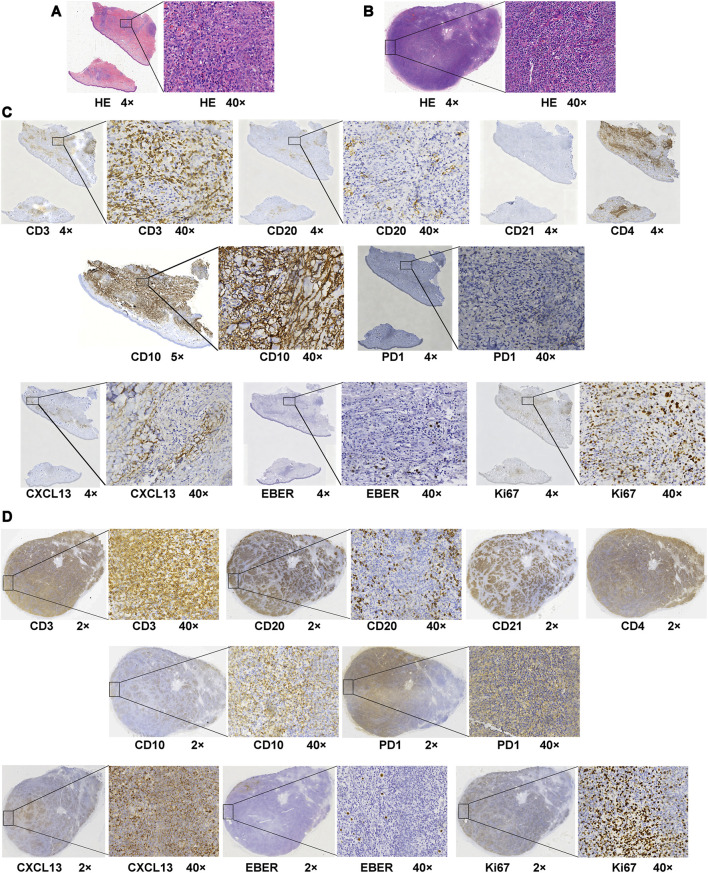
HE findings and IHC results. **(A,B)** HE findings for the skin and lymph node. **(C,D)** IHC findings for the skin and lymph nodes.

**FIGURE 3:**
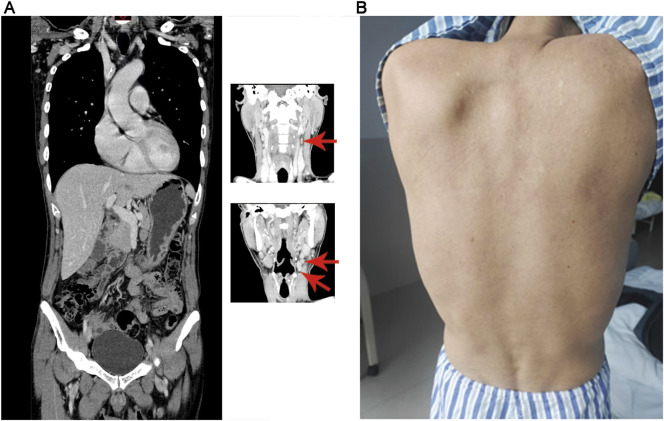
The imaging and cutaneous conditions after chidamide plus CHOP therapy. **(A)** CT demonstrating resolution of the previously noted lymphadenopathy. **(B)** Clinical appearance showing resolution of multiple subcutaneous nodules over the whole body.

On physical examination, multiple subcutaneous nodules measuring approximately 0.5–2 cm in diameter were observed on the back, chest, abdomen and upper limbs. The nodules were moderately firm, non-tender, and covered by dark red infiltrative plaques, without ulceration or purulent discharge (Figure 1A). Laboratory investigations revealed mild anemia, with a hemoglobin level of 112 g/L. The platelet count was 359 × 10^9^/L, and the absolute lymphocyte count was 0.85 × 10^9^/L (11.7%). The white blood cell count was within normal limit. Inflammatory markers were mildly elevated, including C-reactive protein (18 mg/L) and erythrocyte sedimentation rate (35 mm/h). Serum immunoglobulin analysis showed decreased IgA (0.86 g/L) and elevated total serum IgE (198.25 IU/mL) and β2-microglobulin (3.10 mg/L), while IgM and IgG levels were within normal ranges. Autoimmune and infectious screening, including antinuclear antibody (ANA), extractable nuclear antigen (ENA) antibody, antineutrophil cytoplasmic antibody (ANCA), Epstein-Barr virus (EBV), human immunodeficiency virus (HIV), and *treponema pallidum*, were negative. CT imaging revealed multiple mildly enlarged mediastinal lymph nodes, with the largest one measuring approximately 20 mm in short-axis diameter and demonstrating homogeneous contrast enhancement. Moreover, widespread lymphadenopathy was noted in the cervical, supraclavicular, axillary, mediastinal, retroperitoneal, pelvic, and inguinal regions (Figure 1B). Histopathologic examination of an excisional biopsy specimen from the left inguinal lymph node and subcutaneous nodules revealed that the normal architecture of the lymph node is completely interrupted, with destruction of the germinal centers. CD21 immunohistochemistry demonstrates disruption of the follicular dendritic cell meshwork within the lymph node, showing a branching and wind-swept pattern. CD21 is negative in the skin, possibly due to the absence of representative areas of follicular dendritic cell proliferation in the small skin biopsy specimen. The mantle zone is absent. There is marked proliferation of high endothelial venules, exhibiting a branching pattern, with endothelial cell swelling. Basement membrane–like material deposition is observed in the vessel walls and stroma. In the paracortical region and around blood vessels, there is a relatively diffuse infiltration of neoplastic T cells with clear cytoplasm (Figures 2A,B). Immunohistochemical (IHC) analysis was positive for CD3, CD4, CD5, CD10, CXCL13, and Ki67 (40%+), while negative for CD20, CK-pan and CD8 (Figures 2C,D). *In situ* hybridization for EBV-encoded RNA (EBER) was positive in scattered cells. IHC analysis of the subcutaneous nodules yielded similar results with the histopathologic examination (Figure 3). Based on the histopathological and IHC examinations, a diagnosis of AITL was finally established. He was transferred to the hematology department of another hospital for further treatment on September 25, 2025. Pathological review at the referring institution confirmed the diagnosis of AITL, classified as Ann Arbor stage III. For the treatment, the patient received 6 cycles of chidamide plus CHOP, and achieved complete response (CR), with complete resolution of the subcutaneous nodules and superficial lymphadenopathy (Figure 3). The patient was followed up for 11 months with no evidence of recurrence.

## Discussion

AITL is a clinically aggressive subtype of PTCL characterized by LAP, hepatosplenomegaly and B symptoms [5]. Cutaneous involvement has been found in approximately 50% of AITL cases, often manifested by rash and/or pruritus [7]. AITL predominantly presenting with multiple subcutaneous nodules is exceedingly rare [8] and can mimic a variety of conditions including leprosy, connective tissue diseases, and metastatic carcinoma. This case highlights a diagnostically challenging presentation and expands the spectrum of cutaneous manifestations in AITL.

To further characterize the spectrum of dermatologic manifestations, we performed a literature review focusing on rare cutaneous presentations (Table 1). Specifically, Wang et al. reported a case of AITL presenting with dermatomyositis-like skin lesions, in which an elderly man developed widespread erythematous papules over the extensor surfaces of the finger joints, shoulders, and back [9]. Another case report documented a distinctive psoriasiform dermatitis with intermittent trailing scale as the cutaneous presentation of AITL [10]. A case report from Japan described AITL manifesting as erythema multiforme with targetoid lesions and central bullae [11]. Additionally, a case of AITL presenting with agminated miliary facial papules was initially misdiagnosed as reactive B-cell hyperplasia. [12]. Other reported cutaneous manifestations include angioedema [13], prurigo-like lesions [14], and linear IgA bullous dermatosis [15]. Our case is noteworthy in that the initial presentation consisted of multiple subcutaneous nodules without pruritus or rash, posing a significant diagnostic challenge.

**TABLE 1:** A literature review focusing on rare cutaneous presentations in AITL patients.

Author	Age/sex	Past history	Presentation	LAP	LDH	Dermatopathology	Outcome
Wang et al. [9]	80/male	Denied	Diffuse itching, photoaccentuated rash on eyelid, dorsal hand, and back for 6 months	(+)	High (416 IU)	Moderately dense perivascular lymphocytic infiltrate with scattered atypical lymphocytes. IHC: CD3(+), CD10(+), PD-1(+), BCL-6(+), CXCL-13(+), EBER(+)	Died 8 months after diagnosis
McClure et al. [10]	72/male	AITL	Psoriasiform dermatitis	N/A	N/A	Dermal perivascular and focal lobular subcutaneous infiltrate of small lymphocytes and scattered eosinophils. IHC: CD3(+), CD4(+), CD5(+), CD7(+), CD8(+), PD1(+), BCL6(+), CD10(+); CD20(−)	Survived
Hattori et al. [11]	80/female	Gastric ulcer	Erythema multiforme	(+)	High (436 IU)	Scattered epidermal necrotic keratinocytes; dense dermal lymphohistiocytic and granulocytic infiltrates with erythrocyte extravasation and nuclear debris. IHC: CD3(+), CD4(+), CD10(+), Bcl-6(+), PD-1(+); EBER (−)	N/A
Pesqué et al. [12]	59/female	N/A	Agminated miliary facial papules	(+)	N/A	Initial nodular infiltrates evolved into dense perifollicular reactive follicles containing CD3+/CD4+/PD1+/CXCL13+ T cells and scattered EBV + B cells	N/A
Zhao et al. [13]	52/male	Myocardial infarction	Angioedema	(+)	N/A	Normal epidermis with moderate perivascular lymphocytic infiltrate in the dermis and localized vasculotropism. IHC profile: CD4(+), PD1(+), CD10(+), some BCL6(+) and CD21(+). Rare CXCL13(+) cells; Ki-67 approximately 40%	Died 1 year after diagnosis
Shubham et al. [14]	67/male	Hyperpigmented skin lesions	Generalised pruritus with prurigo-like lesions	(+)	N/A	Irregular acanthosis with fused rete ridges, pigment incontinence, and vertical collagen bundles, consistent with prurigo nodularis	N/A
Andriano et al. [15]	80/male	AITL	Linear IgA bullous dermatosis	(+)	N/A	Lymphocytic-eosinophilic interface dermatitis with subepidermal blisters containing neutrophils and eosinophils	N/A
Present case	53/male	Denied	Subcutaneous nodules	(+)	High (327.9)	IHC profile: CD3(+), CD4(+), CD10(+), TIA-1(+), Ki67(+), EBER(+)	Survived

Several differential diagnoses were considered in this case report. Leprosy was initially considered because of the erythema nodosum-like skin lesions; however, the typical clinical features, such as nerve impairment or hair loss, were absent. Connective tissue diseases (e.g., systemic lupus erythematosus, dermatomyositis) may also present subcutaneous nodules. However, they were also excluded due to the negative results of autoantibodies (e.g., ANA, ENA, ANCA) and the absence of systemic manifestations (e.g., myalgia, arthritis). Subcutaneous nodules may also be associated with internal malignancies, including gastric or lung cancer. Nevertheless, the patient had no known history of primary malignancy, and the imaging findings did not reveal a primary tumor.

Notably, cutaneous metastases typically present as firm, isolated nodules, whereas the diffuse infiltrative pattern observed in this case is rarely seen in metastatic disease. While our patient initially presented with cutaneous symptoms, the retrospective history of a long-standing submandibular mass and generalized lymphadenopathy should have raised immediate suspicion for lymphoma. TFH cell-related markers such as PD-1, BCL-6, CXCR5, CXCL13 and ICOS, are often positive and have significant diagnostic value for AITL [16]. However, these markers are not exclusive to AITL and can also be detected in other T-cell lymphomas [17]. Most cases of AITL are diagnosed at an advanced stage because of their nonspecific presentation, as exemplified by the present case. Despite these diagnostic challenges, identifying the consistent expression of PD-1 and CXCL13 in both the lymph node and subcutaneous nodules supported the diagnosis of AITL in this case.

Cutaneous lesions in AITL may represent either reactive immune-mediated manifestations or true neoplastic infiltration secondary to systemic lymphoma involvement [8]. Reactive lesions are thought to result from immune dysregulation and cytokine release, whereas neoplastic lesions usually demonstrate infiltration by TFH phenotype lymphoma cells and are often associated with systemic disease progression [7, 18]. In the present case, the widespread lymphadenopathy, histopathological confirmation of AITL in the lymph node, and the similar immunophenotypic findings in both lymph node and subcutaneous lesions, including PD-1 and CXCL13 expression, favored secondary cutaneous involvement by AITL rather than a purely reactive process. Nevertheless, the absence of molecular clonality analysis in the skin lesion remains a limitation of this study.

Currently, CHOP or CHOEP-based (Etoposide to CHOP) regimens are the standard first-line treatment for AITL, achieving complete remission rates of approximately 40%–60%. Nevertheless, relapse is frequent, and the overall prognosis remains poor, with a 5-year survival rate of approximately 30%–40% [19]. Several emerging therapeutic strategies, including anti-CD30 antibody conjugates, hypomethylating agents (e.g., decitabine), and PD-1 inhibitors, are being explored. However, these agents should be used with caution in clinical practice, as they may be associated with immune-related adverse events. In this study, the patient received 6 cycles of chidamide plus CHOP, and achieved CR with no recurrence during the follow-up.

In summary, we reported a rare case of AITL describing multiple subcutaneous nodules as the primary cutaneous presentation of AITL. Our case expands the clinical spectrum of cutaneous involvement in AITL and highlights previously unrecognized IHC findings.

## Data Availability

The original contributions presented in the study are included in the article/supplementary material, further inquiries can be directed to the corresponding authors.
